# Supplementary material for: Flow Cytometric Assessment of FcγRIIIa-V158F Polymorphisms and NK Cell Mediated ADCC Revealed Reduced NK Cell Functionality in Colorectal Cancer Patients
Source: Cells. 2024 Dec 31;14(1):32. doi: 10.3390/cells14010032 (PMC11720420; doi:10.3390/cells14010032)
Supplement: Supplementary file 1 [file cells-14-00032-s001.zip › cells-3361550-supplementary.pdf]

cells

MDPI

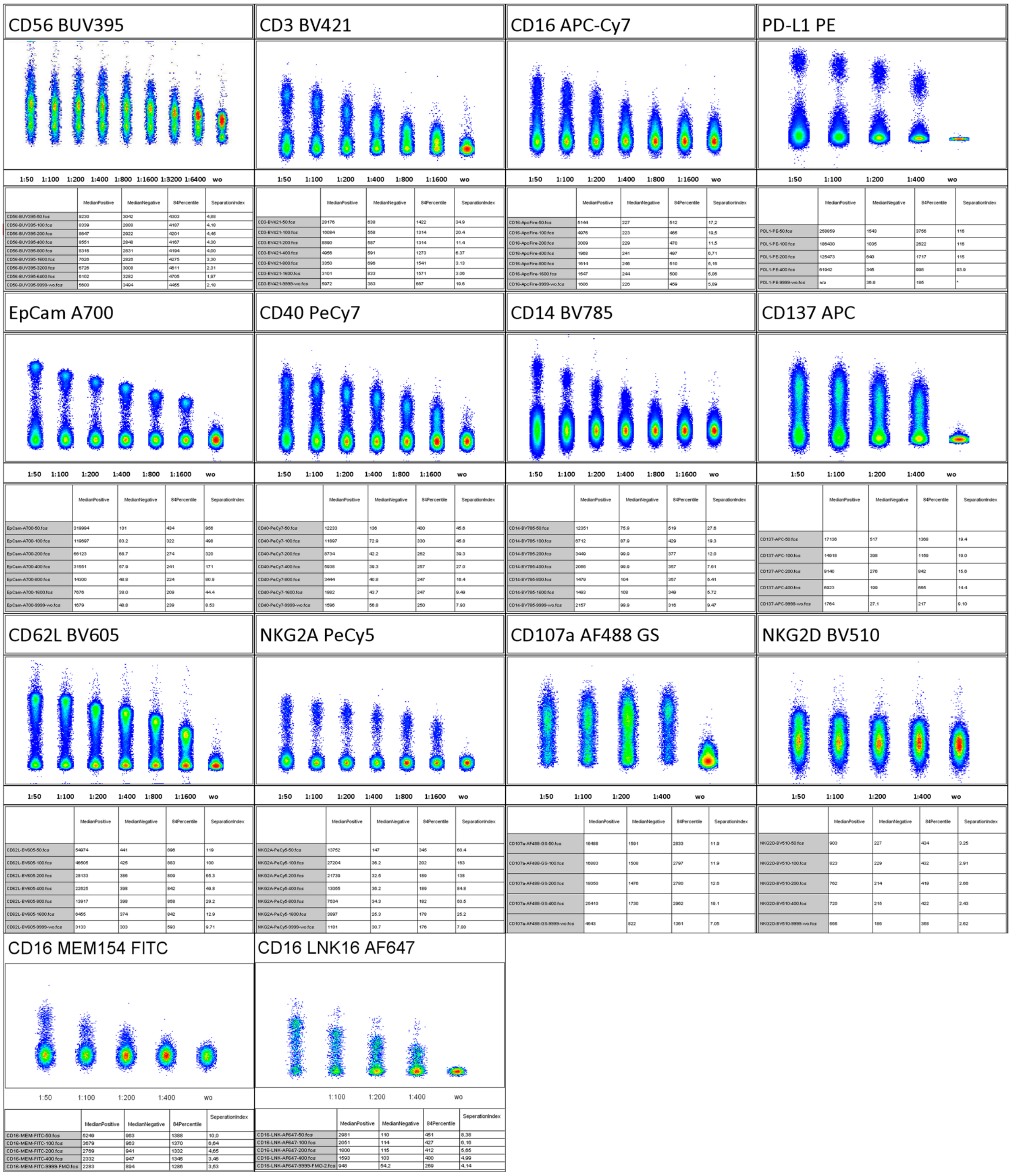

Figure S1. Titration of antibodies for flow cytometry. In total, 5 × 10^5 PBMCs were stained with indicated dilutions of fluorochrome-conjugated antibodies. For titration of EpCAM, PBMCs were mixed at a 1:1 ratio with SNU-C5 cells. Detection of CD137 and CD107a was performed after o.n. stimulation with anti-CD3/CD28 beads (1:1, Miltenyi) and addition of GolgiStop. Optimal antibody dilutions were defined by separation index.

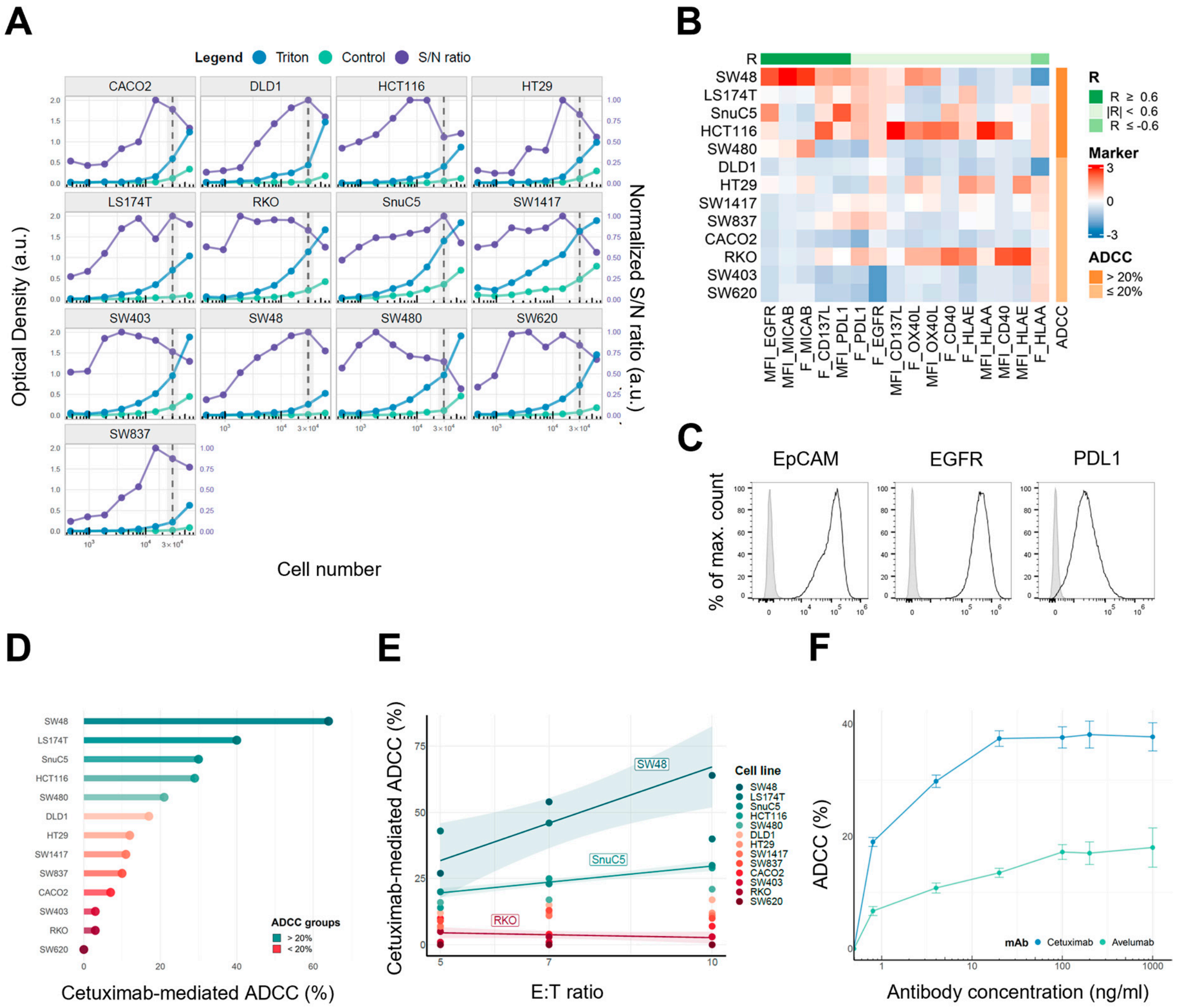

Figure S2. Characterization of 13 colorectal cancer cell lines. (**A**) Detection of signal:noise (S/N) ratios of 13 different CRC cell lines at different cell concentrations. Calculating the ratios between unstimulated samples (control) to the potential maximal release induced by Triton-X-100 revealed a high and stable S/N ratio even for low cell counts. Each dot represents the mean of triplicates. (**B**) All cell lines were stained with PE-conjugated antibodies against the depicted cell surface markers and frequencies of positive cells (F) as well as the MFI of each marker was analyzed. In the heat map, cell lines are ordered according to ADCC sensitivity (see (**D**)) and markers are arranged based on descending Pearson's correlation coefficient (R) between marker expression and ADCC. (**C**) Histograms of EpCAM, EGFR, and PDL1 expression on SNU-C5 cells. The solid black line shows marker expression while the respective isotype control is shown in gray. (**D**) Sensitivity of CRC cell lines to cetuximab-mediated ADCC detected after 24h co-cultures (E:T = 10:1). ADCC was assessed after stimulation with 100ng/mL cetuximab by LDH release assay. Each bar represents the mean of technical triplicates from a representative experiment of at least two individual assays. A cut-off of 20% ADCC was introduced to distinguish cetuximab-resistantfrom sensitive cell lines. (**E**) ADCC sensitivity was also tested at different E:T ratios. While SW48 shows a slightly higher ADCC compared to SNU-C5, their strong adherence in cell culture resulted in high intra-assay variations illustrated by the shaded areas representing the assay standard deviation. (**F**) Titration series of cetuximab and avelumab-induced ADCC against SNU-C5 cells indicates a maximum induced lysis at 100ng/mL. ADCC was determined after 24h at a 10:1 (E:T) ratio by LDH release and each point represents the mean and SEM of a representative experiment.

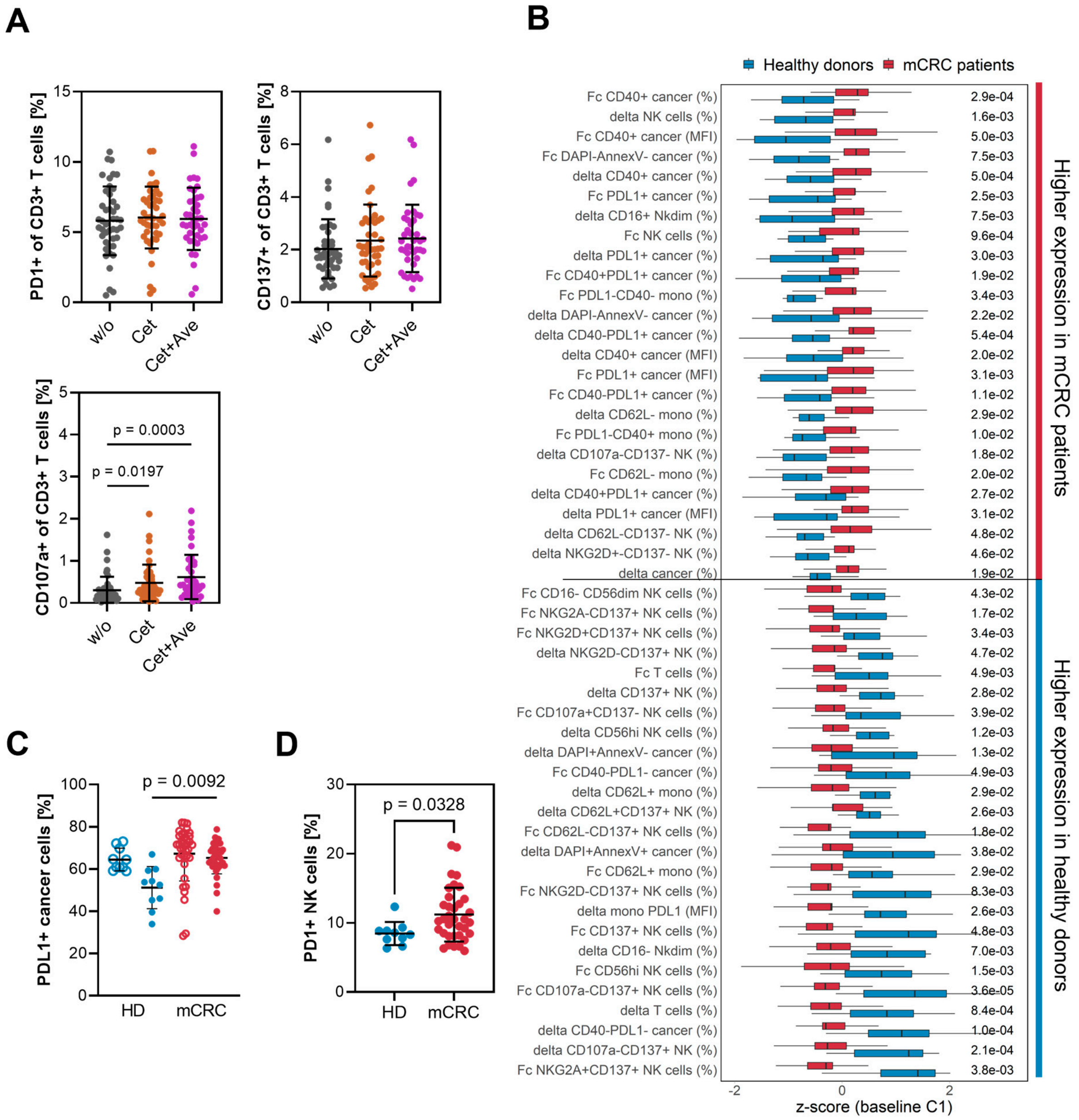

Figure S3. Differentially regulated parameters by cetuximab stimulation. (**A**) Frequencies of PD1, CD137, or CD107a on CD3+ T cells after co-cultures with SNU-C5 cells and stimulation with cetuximab (Cet) alone or in combination with avelumab (Cet+Ave). Data were generated from experiments with 10 HD and 35 mCRC patients and significance was tested by Kruskal–Wallis test. (**B**) Boxplots summarize all significantly differentially regulated parameters after cetuximab stimulation between healthy donors and mCRC patients. Bars show the mean and SEM of z-score normalized values together with the p-value determined by Brunner–Munzel test for non-normal data with unequal variance. Parameters are ordered based on the mean difference between healthy donors and mCRC patients. (**C**) Frequencies of PDL1+ SNU-C5 cancer cells in unstimulated (outlined circles) or cetuximab-stimulated (filled circles) co-cultures with PBMCs from HD or mCRC patients. Statistics were performed using one-way ANOVA followed by Kruskal-Wallis test. D) Frequency of PD1+ NK cells detected in co-cultures without antibody stimulation. Mann–Whitney test was used to test the significance between groups.

Table S1. Study cohort.

Sample ID	Group	Age	Gender	FcyRIIIa
FCY-001	Healthy control	74	male	V/F
FCY-002	Healthy control	70	female	F/F
FCY-003	Healthy control	69	male	V/F
FCY-004	Healthy control	67	female	V/V
FCY-005	Healthy control	68	female	F/F
FCY-006	Healthy control	69	male	V/V
FCY-007	Healthy control	68	male	V/F
FCY-008	Healthy control	68	female	V/F
FCY-009	Healthy control	61	female	V/F
FCY-010	Healthy control	66	male	V/F
FCY-011	Healthy control	38	female	V/V
FCY-012	Healthy control	35	female	V/F
FCY-013	Healthy control	36	male	V/V
FCY-014	Healthy control	35	male	V/F
FCY-015	Healthy control	38	female	V/F
FCY-016	Healthy control	36	male	F/F
FCY-017	Healthy control	34	male	F/F
FCY-018	Healthy control	36	male	V/V
FCY-019	Healthy control	35	female	F/F
FCY-020	Healthy control	33	female	V/F
FCY-021	Healthy control	33	female	V/V
FCY-022	Healthy control	44	female	V/V
FCY-023	Healthy control	42	male	V/V
FCY-024	Healthy control	35	female	V/F
FCY-025	Healthy control	33	male	F/F
FCY-026	Healthy control	26	male	F/F
FCY-027	Healthy control	25	female	F/F
FCY-028	Healthy control	26	female	F/F
FCY-029	Healthy control	48	male	F/F
FCY-030	Healthy control	32	female	F/F
FCY-031	Healthy control	28	female	F/F
FCY-032	Healthy control	26	male	F/F
FCY-033	Healthy control	26	female	V/F
FCY-034	Healthy control	41	female	V/F
FCY-035	Healthy control	49	female	F/F
FCY-036	Healthy control	37	male	V/V
FCY-037	Healthy control	36	male	V/F
FCY-038	Healthy control	31	female	F/F
FCY-039	Healthy control	25	female	V/F
Pat-01	mCRC patient	74	male	F/F
Pat-02	mCRC patient	78	male	F/F
Pat-03	mCRC patient	61	male	F/F
Pat-04	mCRC patient	75	male	F/F
Pat-05	mCRC patient	76	male	F/V
Pat-06	mCRC patient	57	female	F/V
Pat-07	mCRC patient	62	female	F/V
Pat-08	mCRC patient	58	male	F/F
Pat-09	mCRC patient	61	male	F/V
Pat-10	mCRC patient	81	male	F/F
Pat-11	mCRC patient	65	male	F/V
Pat-12	mCRC patient	47	male	F/F
Pat-13	mCRC patient	74	female	F/V
Pat-14	mCRC patient	63	male	F/V
Pat-15	mCRC patient	57	male	V/V
Pat-16	mCRC patient	62	male	F/V
Pat-17	mCRC patient	63	male	F/V
Pat-18	mCRC patient	63	male	V/V
Pat-19	mCRC patient	72	male	F/V
Pat-20	mCRC patient	72	female	F/F
Pat-21	mCRC patient	78	male	F/F
Pat-22	mCRC patient	56	male	V/V
Pat-23	mCRC patient	68	female	V/V
Pat-24	mCRC patient	57	male	F/F

Pat-25	mCRC patient	55	male	F/F
Pat-26	mCRC patient	75	male	V/V
Pat-27	mCRC patient	70	male	F/F
Pat-28	mCRC patient	47	male	F/V
Pat-29	mCRC patient	66	male	F/V
Pat-30	mCRC patient	68	male	F/V
Pat-31	mCRC patient	53	male	F/V
Pat-32	mCRC patient	74	male	F/F
Pat-33	mCRC patient	51	male	V/V
Pat-34	mCRC patient	47	male	F/F
Pat-35	mCRC patient	60	male	F/F
Pat-36	mCRC patient	71	male	F/V
Pat-37	mCRC patient	56	female	V/V
Pat-38	mCRC patient	53	male	F/F
Pat-39	mCRC patient	49	male	F/V
Pat-40	mCRC patient	72	male	F/V
Pat-41	mCRC patient	53	male	F/V
Pat-42	mCRC patient	45	female	F/F
Pat-43	mCRC patient	58	male	F/F
Pat-44	mCRC patient	55	male	V/V
Pat-45	mCRC patient	69	female	F/V
Pat-46	mCRC patient	63	male	F/V
Pat-47	mCRC patient	35	female	V/V
Pat-48	mCRC patient	53	male	F/F
Pat-49	mCRC patient	73	female	F/V
Pat-50	mCRC patient	60	male	F/F
Pat-51	mCRC patient	60	male	F/F
Pat-52	mCRC patient	78	male	F/F

Table S2. Flow cytometry antibodies of the FcγRIIIa panel.

Category	Antibody	Fluorochrome	Clone	Dilution	µl/staining	Company	Catalog No.
Cell lineage	CD3	BV421	UCHT1	1:50	1	Biolegend	300434
	CD14	BV785	M5E2	1:50	1	Biolegend	301840
	CD56	BUV395	NCAM16.2	1:200	0.25	BD Biosciences	563554
FcyRIIIa phenotyping	CD16 MEM	FITC	MEM154	1:50	1	Santa Cruz	sc-51525 FITC
	CD16 LNK	AF647	LNK16	1:50	1	BioRad Antibodies	MCA1193A647T
FcyR block	Beriglobin	-	-	1:50	1	CSL Behring	PZN-046161

Abbreviations: BV, brilliant violet; BUV, Brilliant Ultraviolet; FITC, Fluorescein isothiocyanate; AF, Alexa Fluor.

Table S3. Flow cytometry antibodies for CRC cell line characterization.

Antibody	Fluorochrome	Clone	Dilution	µl/staining	Company	Catalog No.
EpCAM	AF700	9C4	1:400	0.125	Biolegend	324243
EGFR	PE	AY13	1:100	0.5	Biolegend	352903
PDL1	PE	MIH3	1:100	0.5	Biolegend	374512
HLA-A,B,C	PE	W6/32	1:50	1	Biolegend	311406
HLA-E	PE	3D12	1:100	0.5	Biolegend	342603
MICA/B	PE	6D4	1:100	0.5	Biolegend	320906
CD137L	PE	5F4	1:100	0.5	Biolegend	311504
CD40	PE	5C3	1:100	0.5	Biolegend	334308
OX40L	PE	11C3.1	1:100	0.5	Biolegend	326307

Abbreviations: PE, Phycoerythrin; AF, Alexa Fluor.

Table S4. Flow cytometry antibodies of the ADCC panel.

Category	Antibody	Fluorochrome	Clone	Dilution	µl/staining	Company	Catalog No.
Cell lineage	CD3	BV421	UCHT1	1:50	1	Biolegend	300434
	CD14	BV785	M5E2	1:50	1	Biolegend	301840
	CD56	BUV395	NCAM16.2	1:200	0.25	BD Biosciences	563554
	EpCAM	AF700	9C4	1:400	0.125	Biolegend	324243
Checkpoint receptors and activation markers	CD16	APC-Fire750	3G8	1:100	0.5	Biolegend	302060
	CD107a	AF488	H4A3	1:100	0.5	Biolegend	328610
	CD137	APC	4B4-1	1:200	0.25	Biolegend	309809
	CD62L	BV605	DREG-56	1:200	0.25	Biolegend	304834
	NKG2A	Pe-Cy5	S19004C	1:200	0.25	Biolegend	375112
	NKG2D	BV510	1D11	1:50	1	Biolegend	320815
	PD1	BV650	EH12.1	1:100	0.5	BD Biosciences	3564104
Checkpoint ligands	PDL1	Pe-Cy5	MIH3	1:200	0.25	Biolegend	374512
	CD40	Pe-Cy7	5C3	1:200	0.25	Biolegend	374512
Cell death	DAPI	-	-	0.4µM	0.8	Sigma-Aldrich	D9542-5MG
	Annexin V	PeDazzle	-	1:50	1	Biolegend	640956
FcγR block	Beriglobin	-	-	1:50	1	CSL Behring	PZN-046161

Abbreviations: BV, brilliant violet; BUV, Brilliant Ultraviolet; APC, Allophycocyanin; AF, Alexa Fluor; Pe, Phycoerythrin; Cy, Cyanine.

Table S5. Selected parameters to establish a FcγRIIIa prediction model.

Donor	Genotype	Frequency of LNK16+ CD56bright of NK cells [%]	MFI of LNK16 on LNK16+ CD56bright NK cells [MFI]	Frequency of LNK16- CD56bright of NK cells [%]	MFI of LNK16 on LNK16- CD56bright NK cells [MFI]	Frequency of MEM154+ CD56bright of NK cells [%]	MFI of MEM154 on MEM154+ CD56bright NK cells [MFI]	Frequency of MEM154- CD56bright of NK cells [%]	MFI of MEM154 on MEM154- CD56bright NK cells [MFI]	Frequency of LNK16+ CD56dim of NK cells [%]	MFI of LNK16 on LNK16+ CD56dim NK cells [MFI]	MFI of LNK16 on MEM154+ CD56dim NK cells [MFI]	Frequency of LNK16- CD56dim of NK cells [%]	MFI of LNK16 on LNK16- CD56dim NK cells [MFI]	Frequency of MEM154+ CD56dim of NK cells [%]	MFI of MEM154 on LNK16+ CD56dim NK cells [MFI]	MFI of MEM154 on MEM154+ CD56dim NK cells [MFI]	Frequency of MEM154- CD56dim of NK cells [%]	MFI of MEM154 on MEM154- CD56dim NK cells [MFI]	Frequency of LNK16+ of CD56bright NK cells [%]	MFI of LNK16 on CD56bright NK cells [MFI]	Frequency of MEM154+ of CD56bright NK cells [%]	MFI of MEM154 on CD56bright NK cells [MFI]	Frequency of LNK16+ of CD56bright NK cells [%]	MFI of LNK16 on LNK16+ CD56dim NK cells [MFI]	Frequency of MEM154+ of CD56dim NK cells [%]	MFI of MEM154 on CD56dim NK cells [MFI]	Ratio of MFI MEM154/LNK16 on NK cells	Ratio of MFI MEM154 on NK cells/MFI LNK16 on LNK16+ CD56dim NK cells	Ratio of MFI MEM154/LNK16 on CD56dim NK cells	Ratio of Frequency MEM154+/LNK16+ CD56dim NK cells
1	VF	3.07	48372	9.44	1794	2.46	13542	9.86	3638	72.6	62559	10861	14.9	10593	49.7	68186	13684	38	4875	12.5	4078	12.3	4396	87.5	56847	87.7	9395	0.16	0.14	0.22	0.68
2	FF	3.66	44202	21.5	494	3.27	7817	23.1	3681	55.7	55335	3339	19.1	1613	4.36	55743	10542	69.3	3204	25.1	942	26.3	4068	74.9	44202	73.7	3304	0.11	0.06	0.19	0.08
3	VF	2.69	24014	9.39	696	0.92	9481	8.18	3785	68	27430	7909	19.9	3133	39.5	31588	10127	51.4	4653	12.1	1365	9.11	4021	87.9	23177	90.9	6747	0.30	0.23	0.37	0.58
4	VV	1.56	14228	9.24	438	1.68	16301	9.11	3746	75.1	19252	17429	14.1	1790	62.6	20896	19646	26.6	4143	10.8	626	10.8	4214	89.2	17339	89.2	14914	0.82	0.69	1.02	0.83
5	FF	1.85	36060	11.3	644	1.35	6642	12.6	2989	62.2	51042	3169	24.6	2134	5.08	41799	6478	80.9	2970	13.2	979	14	3299	86.8	40403	86	3076	0.09	0.06	0.13	0.08
6	VV	2.35	8769	5.19	954	2	14248	5.89	4520	73.9	15737	14100	18.5	1630	48.9	19029	19135	43.2	4698	7.54	2037	7.89	5669	92.5	12903	92.1	10624	0.82	0.63	1.22	0.66
7	VF	0.39	18037	1.75	324	0.43	9323	1.94	2970	89	32905	9856	8.81	529	68.6	36235	11262	29	4250	2.15	678	2.37	3453	97.9	30327	97.6	9287	0.31	0.28	0.34	0.77
8	VF	0.99	10547	3.52	653	0.54	8395	4.18	3807	82.2	20033	5954	13.2	1074	31.4	25001	8637	63.8	4298	4.51	1180	4.73	4132	95.5	17829	95.3	5498	0.31	0.27	0.43	0.38
9	VF	1.16	23508	3.62	708	0.41	8727	3.53	4010	76.4	32984	6530	18.9	505	31	38585	9132	65.1	4539	4.78	1474	3.94	4208	95.2	27959	96.1	5799	0.21	0.17	0.28	0.41
10	VF	1.94	19707	5.62	232	1.28	7981	5.2	2682	66.5	20508	6495	25.9	286	42.8	21799	7858	50.7	3448	7.56	714	6.49	3228	92.4	15030	93.5	5367	0.37	0.25	0.38	0.64
11	VV	1.63	11427	6.1	244	1.11	17752	7.09	3568	46.2	12417	11088	46.1	417	27.6	16290	16977	64.2	3105	7.73	536	8.2	3930	92.3	4193	91.8	4335	1.18	0.34	1.37	0.60
12	VF	1.93	15521	12.3	339	1.36	8570	13.1	3741	37.4	24014	5520	48.4	523	14.4	31740	10023	71.2	3105	14.2	547	14.4	3924	85.8	4680	85.6	3526	1.12	0.15	0.42	0.39
13	VV	2.17	8750	7.23	600	2.42	12079	7.32	3339	62	15344	14155	28.6	473	50.5	16903	16152	39.7	2903	9.41	1163	9.73	4203	90.6	11225	90.3	9179	0.82	0.53	1.05	0.81
14	VF	2.69	20652	13	751	2.23	9629	11.8	4208	60.8	23287	7587	23.5	1640	29.9	26405	10246	56.1	4796	15.7	1316	14	4685	84.3	18896	86	6360	0.37	0.26	0.44	0.49
15	VF	1.87	14066	7.72	606	1.91	8319	8.7	2880	73.5	22636	7008	16.9	959	43.5	26093	9014	45.9	3851	9.59	1017	10.6	3422	90.4	19893	89.4	6146	0.31	0.26	0.40	0.59
16	FF	0.63	46521	16.9	326	0.42	6327	16.8	1740	72.2	109138	1998	10.2	2556	1.52	66201	6228	81.3	1990	17.6	375	17.2	1771	82.4	101802	82.8	2014	0.02	0.02	0.06	0.02
17	FF	1.65	34115	8.65	538	0.17	6419	10	1865	77.6	73059	2601	12.1	954	3.52	58258	5814	86.3	2534	10.3	886	10.2	1909	89.7	66691	89.8	2601	0.04	0.03	0.08	0.05
18	VV	1.04	13875	6.47	637	1.47	14548	6.31	3930	77.8	20221	16822	14.7	629	66.2	21342	18297	26	4286	7.51	1002	7.78	4539	92.5	18504	92.2	14972	0.80	0.70	0.90	0.85
19	FF	0.99	48609	6.14	896	0.18	7373	7.93	2459	77.6	98814	2764	15.2	1313	0.95	80051	7577	90.9	2745	7.13	1374	8.11	2494	92.9	89704	91.9	2764	0.03	0.03	0.08	0.01
20	VF	1.87	41496	7.36	1210	2.37	10141	6.36	2157	72.2	62252	10206	18.6	1530	63	65391	11088	28.3	3189	9.23	3151	8.73	2880	90.8	54930	91.3	8526	0.16	0.13	0.18	0.87
21	VV	0.72	7460	2.53	379	0.95	10707	2.61	4208	86.3	12168	12157	10.4	1401	67.9	13532	14027	28.5	4570	3.24	763	3.56	5092	96.8	11225	96.4	11160	0.99	0.89	1.15	0.79
22	VV	1.24	14590	4.7	358	1.34	14174	4.79	3584	71.4	18547	15859	22.6	434	58.7	19846	17916	35.2	3406	5.94	696	6.13	4250	94.1	15414	93.9	12001	0.77	0.61	0.97	0.82
23	VV	2.52	14293	5.6	441	2.52	14817	5.79	3981	61.2	20174	16668	30.7	394	50.5	21697	18934	41.2	3370	8.12	1395	8.3	5182	91.9	14825	91.7	10114	0.67	0.46	0.94	0.83
24	VF	1.48	23177	4.13	558	1.03	8471	3.62	2768	64.6	21799	8244	29.8	153	48.8	22214	9654	46.6	2791	5.61	1336	4.65	3406	94.4	15239	95.3	6082	0.40	0.27	0.44	0.76
25	FF	1.19	16982	5	360	0.11	7919	6.56	2569	82.5	36235	2381	11.4	473	0.68	18852	8351	92.7	2360	6.18	676	6.66	2605	93.8	32826	93.3	2373	0.08	0.07	0.23	0.01
26	FF	1.64	22851	1.86	328	0.04	7766	3.8	1897	85.9	23675	1969	10.6	779	0.59	25541	7696	95.6	1913	3.49	3038	3.84	1897	96.5	20652	96.2	1925	0.10	0.08	0.33	0.01
27	FF	1.02	54394	5.65	1188	0.27	8727	6.4	4457	79.7	90150	4274	13.7	896	3.02	86218	8604	90.3	4108	6.67	3164	6.67	4558	93.3	83076	93.3	4185	0.05	0.05	0.10	0.04
28	FF	5.59	28093	12.6	531	1.45	8033	15.1	4323	67.7	45846	3768	14.1	974	4.76	49810	8233	78.7	3675	18.2	1724	16.6	4495	81.8	41901	83.4	3790	0.11	0.09	0.18	0.07
29	FF	3.81	53470	5.89	3603	0.39	8022	9.02	4167	86.3	73602	3719	3.98	9140	2.15	80846	8255	88.4	3670	9.69	19478	9.41	4262	90.3	72521	90.6	3703	0.05	0.05	0.11	0.02
30	FF	1.69	67351	5.29	1498	0.25	7898	6.48	4191	88.5	91274	3958	4.54	1549	2.13	92641	8637	91.1	3902	6.99	7508	6.74	4226	93	89260	93.3	3941	0.05	0.04	0.09	0.02
31	FF	2.35	45512	7.19	1851	1.14	8471	7.76	4602	78.5	59123	4149	12	318	7.69	60891	9191	83.4	3840	9.54	5105	8.9	4902	90.5	55607	91.1	4039	0.08	0.07	0.16	0.10
32	FF	3	30988	24.5	1700	1.67	9073	25.1	4717	62.5	50176	4085	10	6068	4.49	57267	10875	68.7	3835	27.5	2551	26.8	4888	72.5	45846	73.2	3953	0.11	0.08	0.22	0.07
33	VF	4.22	25969	12.5	651	3.84	9120	13	4021	72.2	33869	6042	11	1237	25.2	39150	8933	57.9	4756	16.8	1956	16.9	4724	83.2	31212	83.1	5722	0.19	0.16	0.26	0.35
34	VF	3.94	44634	17.7	3710	8.03	10233	13.9	4615	72.9	64750	9831	5.48	8146	55.9	67186	10931	22.2	5791	21.6	6068	21.9	6098	78.4	63177	78.1	9567	0.16	0.14	0.17	0.77
35	FF	0.83	48372	4.27	389	0.2	8933	5.1	3998	82.2	67851	4316	12.7	265	4.32	70929	8693	90.4	4021	5.1	680	5.3	4114	94.9	63489	94.7	4138	0.07	0.06	0.13	0.05
36	VV	0.65	29117	2.56	1458	1.26	23616	2.59	5381	89.4	33303	22754	7.43	3943	81.7	33869	23585	14.4	7230	3.21	2713	3.86	7656	96.8	32279	96.1	21809	0.67	0.65	0.71	0.91
37	VF	1.81	43988	20.6	394	2.94	8085	19.5	3174	59	48490	6090	18.6	1855	29	52691	8002	48.6	4085	22.4	569	22.4	3437	77.6	43245	77.6	5359	0.13	0.10	0.17	0.49
38	FF	2.87	36586	17.9	488	0.99	8945	15.8	4621	72.3	63177	3896	6.91	1068	1.99	58116	8637	81.2	3812	20.8	754	16.8	4763	79.2	60444	83.2	3868	0.08	0.06	0.14	0.03
39	VF	1.13	40013	8.07	959	2.37	9311	6.99	4179	82.2	62405	8649	8.55	5472	62.3	65230	9667	28.3	5505	9.2	1470	9.36	4989	90.8	60444	90.6	8308	0.14	0.13	0.15	0.76

Abbreviations: MFI, Median fluorescent intensity.

Table S6. Characteristics of colorectal cancer cell lines.

Cell Line	Disease	Medium	Additives	RRID
CACO2	Colorectal adenocarcinoma	DMEM	10% FBS, 1% P/S, 1% GlutaMax	CVCL_0025
DLD1	Dukes' type C, colorectal adenocarcinoma	RPMI1640	10% FBS, 1% P/S, 1% GlutaMax	CVCL_0248
HT29	Colorectal adenocarcinoma	DMEM	10% FBS, 1% P/S, 1% GlutaMax	CVCL_0320
HCT116	Colorectal adenocarcinoma	DMEM	10% FBS, 1% P/S, 1% GlutaMax	CVCL_0291
LS174T	Dukes' type B, colorectal adenocarcinoma	DMEM	10% FBS, 1% P/S, 1% GlutaMax	CVCL_1384
RKO	Carcinoma, papilloma	DMEM	10% FBS, 1% P/S, 1% GlutaMax	CVCL_0504
SNU-C5	Cecum adenocarcinoma	RPMI1640	10% FBS, 1% P/S, 1% GlutaMax	CVCL_5112
SW48	Dukes' type C, colorectal adenocarcinoma	Leibovitz's L-15	10% FBS, 1% P/S, 1% GlutaMax	CVCL_1724
SW403	Dukes' type C, colorectal adenocarcinoma	Leibovitz's L-15	10% FBS, 1% P/S, 1% GlutaMax	CVCL_0545
SW480	Dukes' type B, colorectal adenocarcinoma	Leibovitz's L-15	10% FBS, 1% P/S, 1% GlutaMax	CVCL_0546
SW620	Dukes' type C, colorectal adenocarcinoma	Leibovitz's L-15	10% FBS, 1% P/S, 1% GlutaMax	CVCL_0547
SW837	Colorectal adenocarcinoma	Leibovitz's L-15	10% FBS, 1% P/S, 1% GlutaMax	CVCL_1729
SW1417	Dukes' type C, colorectal adenocarcinoma	Leibovitz's L-15	10% FBS, 1% P/S, 1% GlutaMax	CVCL_1717

Abbreviations: RRID, Research Resource Identifier; FBS, Fetale bovine serum; P/S, Penicillin-Streptomycin.
